# Secular trends in 1,192 diarrheal outbreaks in the Israel Defence Forces between 1988–2011

**DOI:** 10.1186/s40696-015-0004-1

**Published:** 2015-06-08

**Authors:** Sharon Elazar, Yifat Zelikovich, Hagai Levine, Anat Tzurel-Ferber, Inbal Galor, Michael Hartal

**Affiliations:** 1 Israel Defense Forces Medical Corps, Department of Public Health, Tel Aviv, Israel; 2 Hebrew University-Hadassah Braun School of Public Health, Jerusalem, Israel; 3 Department of Military Medicine, Hebrew University Faculty of Medicine, Jerusalem, Israel

**Keywords:** Diarrheal-outbreaks, Summer, Military, Training

## Abstract

**Background:**

In the military, outbreaks of infectious diarrhea pose a significant health problem. In this descriptive analysis of data collected by the IDF on all infectious diarrhea outbreaks between 1988–2011, we analysed temporal, seasonal, and unit-type trends in 1,192 diarrheal outbreaks in the Israel Defence Forces (IDF) over a 24-year period, and described the long-term trends in seasonality and the effects of strategic preventive measures on outbreak frequency among populations at risk.

**Results:**

We found two distinct phases in annual outbreak occurrence. The mean annual number of outbreaks during the period 1988–1996 was 75.8 (*±*14.50) but dropped to 34.0 (*±*8.13) during the period 1997–2011 (P < 0.0001). Overall, a downward trend continued through the 1990’s, while from 2000 onwards outbreak counts fluctuated annually. A significantly higher number of outbreaks occurred during the summer season, throughout the study period. The greatest number of outbreaks occurred in deployed units, although the proportion of outbreaks in this unit type decreased over time. Accordingly, the proportion of outbreaks in training units more than doubled during the study window. When we looked at outbreak size, summer outbreaks increased in magnitude over time, and during all periods outbreaks were larger, on average, in training units than in deployed units.

**Conclusions:**

The changing patterns in diarrheal outbreaks in the Israel Defence Forces require maintenance of a higher level of vigilance than ever before. Lack of a clear peak period require the use of all available preventive measures throughout the year. This is especially true in training units, where the increased number of outbreaks coincides with increased trainee volume, regardless of season.

## Background

Infectious diarrhea remains a significant health problem, potentially leading to considerable morbidity, lost workdays and significant economic costs [1]. In the military, such outbreaks have been known to incapacitate large numbers of soldiers [2,3] in active-duty units such as flight staff [4], naval crews [5] and soldiers on training bases [6]. Risk factors for diarrheal outbreaks in military units are crowded living conditions, sub-standard personal and unit hygiene practices, mass food processing facilities, lack of adequate refrigeration under field conditions, and frequent movement of personnel among units and throughout various geographic locations [1].

In wars fought during the first half of the 20th century, diarrhea was the most common source of morbidity due to infectious disease. More recently, large outbreaks have been reported among armies operating in Afghanistan and Iraq [3,4,7-11]. Diarrheal outbreaks and cases of food poisoning have been described in numerous other armies as well, throughout Europe, Africa and the Middle and Far East [1,12-15].

Within the Israel Defence Force (IDF), infectious diarrhea, involving sporadic illness and outbreaks, has been and still remains a public health problem. Previous authors have described the epidemiological characteristics of food-borne outbreaks in the IDF, finding that the overall incidence of outbreaks decreased over time, as did the number of soldiers involved in any given outbreak. [1,15,16] However, these earlier studies did not examine the seasonal distribution of outbreaks, and they studied relatively short periods of time, thus preventing them from observing long-term trends in outbreak characteristics. The purpose of the current report is to extend the epidemiological analysis of diarrheal outbreaks through 2011, while describing the long-term trends in seasonality and the effects of strategic preventive measures on outbreak frequency among populations at risk.

## Methods

This study was a retrospective analysis of epidemiological data gathered by the IDF Medical Corps’ Army Health Branch, representing all reported outbreaks of diarrhea in the Israeli military between 1988 and 2011. Military regulations mandate reporting of all suspected outbreaks of gastroenteritis in a military unit. A suspected outbreak is declared when at least five soldiers in a single unit develop diarrhea within one 24-hour period, or when eight or more soldiers develop diarrhea over a 48-hour period. Epidemiologic investigation is required when an outbreak has been confirmed on the basis of one of the following definitions: (a) ten or more soldiers in a single unit develop diarrhea in a single day; (b) 15 or more soldiers in a single unit develop diarrhea over a two-day period; (c) 25% of the personnel in a single unit develop diarrhea within a seven-day period.

The 24 year study window was divided into six 4-year time periods for subanalysis. The summer season was defined as the period between April 1 and October 31 of each year, and the winter season as November 1 through March 31 of the following year. Military unit types were classified as “deployed” (e.g. battalions, brigades and other line units), “training” (e.g. basic training camps, advanced training bases) and “garrisoned” (e.g. headquarters, offices).

Data were collected routinely throughout the study period and stored using relational database software (Microsoft® Access). We used the nonparametric Mann-Kendall test for monotonic trend to test for changes in outbreak frequency and size over time. We compared seasonal and unit-type outbreak distributions between time periods using x^2^ tests. Graphs were created using Microsoft® Excel.

## Results

**Figure 1:**
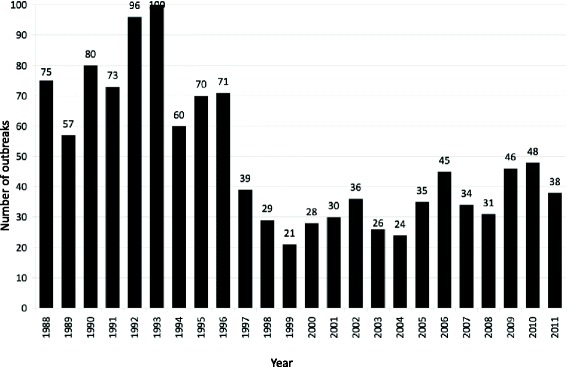
Annual number of diarrhea outbreaks in the IDF, 1988–2011 (N = 1,192).

A total of 1,192 outbreaks were reported over the 24-year study period. Figure 1 illustrates the number of outbreaks by year. The greatest number of outbreaks (100) occurred in 1993 and the nadir (21) was observed in 1999. The figure shows two distinct phases in annual outbreak occurrence. The mean annual number of outbreaks during the first phase (1988–1996) was 75.8 *±* 14.50 but dropped to 34.0 *±* 8.13 during the second phase (1997–2011, P < 0.0001). Overall, a downward trend continued through the 1990’s, while from 2000 onwards outbreak counts fluctuated annually.

**Figure 2:**
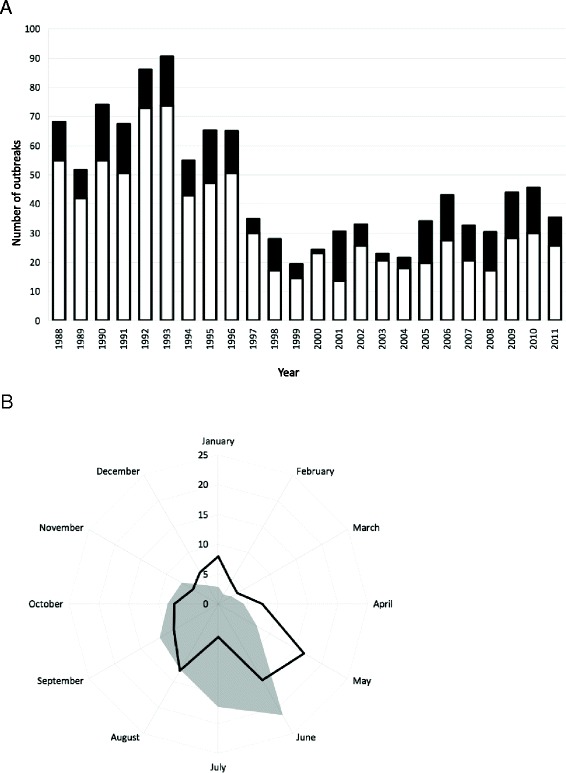
Seasonal distribution of IDF diarrhea outbreaks. Panel **(A)**: distribution by summer (white)/ winter (black), adjusted for season length. Panel **(B)**: distribution by month for periods 1 (1988–1991, shaded) and 6 (2008–2011, non-shaded).

We analyzed the number of outbreaks by season. Figure 2A shows a higher number of diarrhea outbreaks in the summer time compared to winter. The data presented in this figure have been adjusted for the unequal season lengths in Israel (7 months for summer, 5 months for winter). The strong preponderance of outbreaks during the summer season remained constant over the entire study period, despite the overall decrease over time in the total number of annual outbreaks. We further explored the distribution of outbreaks by month (Figure 2B). In the earliest years (period 1, 1988–1991) the frequency of outbreaks peaked in the month of June. During this period, 21.4% of all outbreaks occurred in June, and on average, 51% of the annual outbreaks occurred during the months June-August. In contrast, during the latest years (period 6, 2008–2011) this pattern changed, and the bulk of outbreaks occurred earlier in the year. During period 6, outbreak frequency peaked in the month of May (16.6%), and the bulk (38.6%) of the outbreaks occurred over an earlier 3-month period between April and June. The reduction in the proportion of outbreaks during the peak summer season (June-August) was statistically significant (P < 0.001). Overall, the monthly distribution of outbreaks was more seasonally polarised during period 1 (more in summer months, fewer in winter months), and more equally distributed in period 6, with a reduction in the relative frequency during the summer months (May-October), and an increase in the relative frequency during the winter months (November-April).

**Figure 3:**
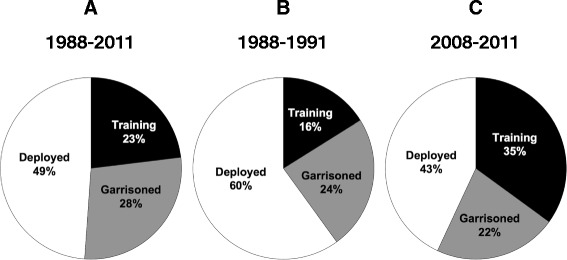
Distribution of diarrheal outbreaks by unit type and period. Panel **(A)** 1988–2011; panel **(B)** 1988–1991; panel **(C)** 2008–2011.

We examined the distribution of outbreaks by unit type over time. We found that over the entire study period, 49% of the outbreaks occurred in deployed units, 23% in training units and 28% in garrisoned units (Figure 3, panel A). This pattern, however, changed significantly over time. During period 1 (1988–1991) 60% of outbreaks occurred in deployed units, but this proportion decreased to 43% by period 6 (2008–2011, P = 0.001) (Figure 3, panels B and C). Accordingly, the proportion of outbreaks in training units more than doubled during the study window, from 16% in period 1 to 35% in period 6 (P < 0.001). The proportion of outbreaks in garrisoned units remained unchanged (24% in period 1, 22% in period 6, P = 0.67).

The mean number of cases per outbreak is shown in Table 1, by period and season. Summer outbreaks increased in size over time, from a mean of 34.1 cases per outbreak during period 1 to 42.1 during period 6 (Mann-Kendall test for trend P < 0.02). There was no increase over time in the mean size of winter outbreaks or in the ratio of summer/winter outbreak size (P > 0.2 for both tests). Table 2 shows the mean number of cases per outbreak by period and unit type. There was no apparent trend in outbreak size over time in any of the unit type strata. However, in all periods outbreaks were larger, on average, in training units than in deployed units (40%-112% more cases per outbreak); this difference in mean outbreak size was present but less pronounced in the comparison to garrisoned units.

**Table 1:** **Mean number of cases per outbreak by season and period**

**Period**	**Years**	**Summer**	**Winter**	**r**_**summer/winter**_
1	’88-’91	34.1	35.7	0.96
2	’92-’95	34.5	21.2	1.63
3	’96-’99	34.7	35.4	0.98
4	’00-’03	40.9	27.7	1.48
5	’04-’07	35.6	43.3	0.82
6	’08-’11	42.1	39.0	1.08

(*r*-ratio).

**Table 2:** **Mean number of cases per outbreak, by unit type and time period**

**Period**	**Years**	**Field of service**	**Field of training**	**Non-field**	***r***_**FT/FS**_	***r***_**FT/NF**_
1	’88-’91	30.2	45.1	37.6	1.49	1.20
2	’92-’95	26.8	44.3	35.4	1.65	1.25
3	’96-’99	26.8	48.6	36.7	1.81	1.32
4	’00-’03	31.1	43.5	43.2	1.40	1.01
5	’04-’07	26.8	56.8	36.1	2.12	1.57
6	’08-’11	29.6	60.0	36.5	2.03	1.64

(*r*-ratio).

FT - Field trainings, FS - Field service units, NF-non-field units.

T – training units, D – deployed units, G – garrisoned units.

## Discussion and conclusions

In this study we analysed temporal and seasonal trends in 1,192 diarrheal outbreaks in the IDF over a 24-year period. We found two distinct phases with a dramatic decrease in the mean annual number of outbreaks from 1997 onwards. Overall, a downward trend continued through the 1990’s, while from 2000 onwards outbreak counts fluctuated annually. This observation underscores previous findings [1,15] and shows that the downward trend in annual incidence has not continued into the first decade of the 21st century. Although some year-to-year variability is present, it appears that the annual incidence of diarrheal outbreaks has remained stable over the last decade. Previous reports [1,15], discussed interventions that may provide some explanation of these observations. These include improvements in infrastructure, early outbreak identification and rapid intervention and strict personal, unit-level and environmental hygiene regulations, all of which have been embedded in routine IDF outbreak prevention policy. Since there were no major changes in our overall population size in the last two decades, a smaller population-at-risk is not a plausible explanation for the pronounced decrease in the annual number of outbreaks observed since the mid 1990’s. This trend, however, may be attributed to changes in the geographic repositioning of many army units from older bases to newer, larger camps with improved infrastructure. This hypothesis is supported by a post-hoc qualitative analysis of our data, which showed that many of the outbreaks in the early 1990’s occurred on bases decommissioned during this redeployment process.

We compared our military data to those available for the overall national population. In Israel, the frequency of infectious diarrhea outbreaks remained stable over the period of interest, ranging from 28–64 outbreaks annually, with a single peak of 93 outbreaks in 2004 [17]. The national Israeli data, which do not include military reports, showed no evidence of the dramatic drop in frequency observed in the army data after 1996; similarly, the military data did not show the frequency spike in 2004 observed in the national data. Additionally, pathogen-specific epidemiologic data from national registries further underscore the differences in overall population morbidity patterns compared to our military findings. There was a clear cyclic pattern in the national annual incidence rate of Shigella cases during the study period, with peaks occurring every 2–3 years [18,19]. While our study looked at all-pathogen outbreak counts rather than pathogen-specific incidence rates, a cyclic pattern was nonetheless conspicuously absent in our setting. Together, these observations seem to indicate that patterns in outbreak frequency in the IDF are due to changes intrinsic and unique to the military environment and are independent of parallel trends in the overall population, although the decrease in the incidence of sporadic Shigella cases in Israel over time [19], especially among children [18], may indicate that Israeli soldiers have, in recent years, become more susceptible to gastrointestinal pathogens than in the past.

A prominent finding of this study is the marked seasonal distribution of outbreaks, with a preponderance of outbreaks occurring during the summer months. This trend remained even after adjusting for the longer summer season and shorter winter season characteristic of Israel's climate. Summer months are characterised by hotter temperatures, more daylight hours, and, in the military setting, more field-based activities carried out under conditions of compromised personal and environmental hygiene. In Israel, this is also a time of significantly increased fly activity, which has been shown to be directly associated with higher rates of sporadic and epidemic diarrhea. [16,20-22]. Housefly densities vary with temperature, number of sunshine hours, humidity and availability of breeding sites. In tropical and subtropical climates, fly density increases as mean daily temperature rises following the end of the cool season; however, as mean daily temperatures approach their peak in the hot season, housefly density then decreases [20]. Fly control as a public health intervention, largely based on bait and trap strategy, has been demonstrated to greatly reduce the prevalence of houseflies, and cohorts of Israeli soldiers trained on bases where intensive fly control measures were implemented were shown to have had significantly less diarrheal disease [20]. Fly control measures, as well as additional preventive interventions such as increased surveillance, inspections and commander awareness, were concentrated during the summer months, likely explaining the decrease in frequency during this season observed over our study period. While the bulk of outbreaks in the earlier periods clearly occurred during the months of June-August, the proportion of outbreaks during these months is now smaller than in the past. This finding was statistically significant.

We observed clear trends related to unit type as well. There was a 28% relative reduction in the proportion of outbreaks in deployed units during the study period. This trend coincided with a major redeployment and redistribution of deployed units that occurred during this period, during which many small and medium-sized units were combined and relocated to larger bases with improved infrastructure. Conversely, there was a relative increase of 118% in the proportion of outbreaks in training units over time. Furthermore, on average, outbreaks on training bases were larger than those in other unit types. These observations are likely attributable to the IDF’s shift to larger training cohorts during the study period, with more soldiers being exposed to the crowding, compromised personal hygiene, challenging environmental conditions and strained infrastructure that are characteristic of these bases during peak training periods. Thus, when outbreaks occurred in these units, they tended to be of a larger magnitude.

One potential limitation of studies based on notifiable disease data is their sensitivity to reporting compliance and completeness. In the US, during the period 1998–2008, the number of foodborn disease outbreaks reported annually was more than double that reported during the years 1973–1997. This dramatic increase has been attributed mainly to the effects of increased surveillance rather than a true increase in outbreak frequency [23]. Similarly, a study conducted in Italy demonstrated significantly higher notification rates of food borne disease outbreaks after the implementation of a new surveillance system [24]. This, however, is likely not a major limitation of the current study. Notifiable disease reporting is highly regulated and controlled in the army setting. Report rates and completeness are stable and compliance is enforced based on military rules and regulations. Furthermore, the clear annual patterns of military outbreak frequency seem to indicate a true seasonal difference in morbidity, while reporting compliance is unlikely to be seasonally dependent.

The changing patterns in diarrheal outbreaks in the IDF require us maintain a higher level of vigilance than ever before. If, in the past, preventive measures could be concentrated on the “peak summer months” (June-August), today’s more homogenous spread over the entire warm season require the use of all available preventive measures throughout the year.

## Abbreviation

IDF: Israel Defence Forces
